# Long noncoding RNA FAM83A-AS1 facilitates hepatocellular carcinoma progression by binding with NOP58 to enhance the mRNA stability of FAM83A

**DOI:** 10.1042/BSR20192550

**Published:** 2019-11-12

**Authors:** Jinyu He, Jiao Yu

**Affiliations:** 1 Department of Hepatology, Shaanxi Traditional Chinese Medicine Hospital, Xi’an 710000, Shaanxi, China; 2 Department Two of Splenolopy and Gastrology, Shaanxi Traditional Chinese Medicine Hospital, No.2 Xihua Men, Xi’an 710000, Shaanxi, China

**Keywords:** FAM83A-AS1, FAM83A, hepatocellular carcinoma, NOP58

## Abstract

Hepatocellular carcinoma (HCC), as one of the commonest cancers globally, is a primary malignancy in human liver with a characteristic of high mortality rate. Long noncoding RNAs (lncRNAs) are confirmed to be implicated with multiple cancers including HCC. LncRNA FAM83A-AS1 has also been validated as an oncogene in lung cancer, but its mechanism in HCC is poorly understood. Our research is intended to investigate the underlying mechanism of FAM83A-AS1 in HCC. In the present study, we found the abundantly increased expression level of FAM83A-AS1 in HCC tissues and cells. FAM83A-AS1 inhibition hampered cell proliferation, migration and elevated cell apoptosis in HCC. Moreover, FAM83A-AS1 could positively regulate FAM83A, and FAM83A could also promote the progression of HCC. In addition, FAM83A-AS1 and FAM83A were both verified to bind with NOP58, and FAM83A-AS1 enhanced the mRNA stability of FAM83A by binding with NOP58. In rescue assays, the suppressed influence of down-regulated FAM83A-AS1#1 on cell proliferation, migration as well as the accelerated influence of FAM83A-AS1#1 knockdown on cell apoptosis could be partially recovered by overexpression of FAM83A. In conclusion, FAM83A-AS1 facilitated HCC progression by binding with NOP58 to enhance the stability of FAM83A. These findings offer a novel biological insight into HCC treatment.

## Introduction

Hepatocellular carcinoma (HCC), as one of the most common cancers, is the leading cause of cancer-associated deaths in China with a characteristic of high morbidity and mortality [1–3]. One sorrowful fact about HCC is that the number of HCC patients has been increasing annually. Accumulating researches have been done to uncover the main risk factors of HCC, showing that HCC is mainly induced by alcoholism, hepatitis B virus or hepatitis C virus infection, liver cirrhosis and so on [4]. Although progress about potentially curative treatments for HCC was achieved in the past few years, the outcomes of patients with HCC still remain unsatisfactory with the 5-year survival rate under 30% [5,6]. Therefore, it is imperative to take the specific underlying mechanisms in HCC into consideration, which might provide new biomarkers and novel therapeutic strategies for HCC prognosis and treatment.

Long noncoding RNAs (lncRNAs) serve as pivotal components of non-coding RNAs with over 200 nucleotides (nt) in length with absence of coding protein potential [7]. LncRNAs are certified to participate in human genome and appeal to researchers due to their specific function in different diseases [8]. Growing evidence has exposed us to lncRNAs that are implicated with multiple cancers. For example, lncRNA ATB promotes malignant melanoma progression by sponging miR-590-5p to enhance the expression of YAP1 [9]. LncRNA AFAP1-AS1 is up-regulated in colon cancer and accelerates colon cancer progression [10]. LncRNA SNHG15 was an oncogene in ovarian cancer to promote cell proliferation, migration and invasion in epithelial ovarian cancer cells [11]. LncRNA LINC01296 activates cancer cell proliferation and metastasis to enhance the progression of urothelial carcinoma of the bladder [12]. A recent study found that lncRNA FAM83A-AS1 acted as an oncogenic gene to promote the progression of lung cancer [13]. Although the biological function of FAM83A-AS1 in lung cancer has been clearly classified, whether it exerts a role in the same pattern in HCC has not been well elucidated.

Our current research focused on exploring whether FAM83A-AS1 participated in the development of HCC. Our research proved that FAM83A-AS1 facilitated HCC progression by binding with NOP58 to enhance the mRNA stability of FAM83A. This finding might serve as an effective clue to identify novel ceRNA mechanism for HCC research.

## Materials and methods

### Tissue samples

HCC and adjacent non-tumor tissues were collected from HCC patients who were validated by histopathological evaluation in Shaanxi Traditional Chinese Medicine Hospital. None of the patients received chemotherapy or radiation therapy before they underwent the operation. All collected tissue specimens were immediately frozen and stored in liquid nitrogen until required. The present study was approved by the Research Ethics Committee of Shaanxi Traditional Chinese Medicine Hospital with written informed consent obtained from all patients.

### Cell culture

Four human HCC cell lines (SMMC-7721, HepG2, HuH-7, H-97) and human normal liver cell line (LO2) were purchased from the Shanghai Cell Bank of the Chinese Academy of Sciences. All the cells were stored in Dulbecco’s modified Eagle medium (DMEM) (Thermo Fisher Scientific, Waltham, MA, U.S.A.) consisting of 10% fetal bovine serum (FBS) (Corning, NY, U.S.A.) and 1% penicillin/streptomycin (Life Technologies, Waltham, MA). Cells were cultured in a 5% moist air incubator at 37°C.

### Cell transfection

Lipofectamine 2000 (Invitrogen, U.S.A.) was applied for performing the transfections. FAM83A overexpression vector was structured by GenePharma (Shanghai, China). Three FAM83A-AS1 shRNAs (sh-FAM83A-AS1#1/2/3), FAM83A shRNAs (FAM83A#1/2/3), NOP58 shRNA and respective scrambled negative control shRNA (sh-NC) were gained from Invitrogen (Carlsbad, CA, U.S.A.).

### Quantitative real-time polymerase chain reaction

TRIzol reagent (Invitrogen) was employed to extract the total RNAs from tissues and cells. Complementary DNAs (cDNAs) emerged by use of the PrimeScript RT reagent kit (Takara, Japan). Quantitative real-time polymerase chain reaction (qRT-PCR) was conducted with SYBR-Green Real-Time PCR Kit (Takara, Otsu, Japan). GADPH or U6 acted as the internal control. The relative expression levels were calculated by the method of 2^−ΔΔ*C*_t_^.

### Cell proliferation assay

CCK-8 assay was utilized to estimate the proliferation rates of HCC cells with CCK-8 kit (Dojindo, Kumamoto, Japan). Cells were incubated in 96-well plates for 0, 24, 48, 72 and 96 h. CCK-8 solution was filled in each well daily and incubated with the cells for 2 h. Finally, cell viability was measured with a microplate reader at the absorbance at 450 nm.

### Colony formation assay

The colony formation ability of HCC cells was measured by colony formation assay. Briefly, HCC cells were seeded in six-well plates after transfection. Two weeks later, colonies were fixed with methanol, and stained with 0.1% Crystal Violet (Sigma–Aldrich). The number of stained colonies was observed and counted using a phase-contrast microscope.

### Transwell assay

HCC cells migration ability was evaluated by transwell assay. Briefly, cells resuspended in serum-free DEME medium were planted into the top chamber. Medium supplemented with 10% FBS was filled in the lower chamber. Cells were then cultured in the chamber for 24 h and migrated cells were dyed with 0.1% Crystal Violet (Sigma–Aldrich). Finally, the number of migrated cells in random fields was counted under a microscope (Leica, Wetzlar, Germany).

### RNA immunoprecipitation assay

RNA immunoprecipitation (RIP) was performed to probe whether FAM83A-AS1 and FAM83A could bind with NOP58. Magna RIP RNA Binding Protein Immunoprecipitation Kit (Millipore, Billerica, MA, U.S.A.) was used for the assay. Lysed with complete RIP lysis buffer, HCC cells were incubated with RIPA buffer ligated to human anti-Argonaute (anti-Ago2) or anti-IgG (Millipore). Finally, qRT-PCR was performed to analyze the expression of purified RNA.

### Western blot

HCC cells were lysed with RIPA lysis solution (Beyotime). Afterward, protein lysis was separated by 10% SDS/PAGE electrophoresis and then transferred on to a PVDF membrane (Millipore). The membrane was blocked with 5% skimmed milk followed by incubating with the primary antibodies. All the primary antibodies were purchased from Abcam (Cambridge, U.K.) including anti-Bcl-2 (ab32124), anti-Bax (ab32503), anti-Cleaved caspase3 (ab2302), anti-caspase3 (ab13847) and anti-GAPDH (ab9485). Then secondary antibody was used to incubate the membrane. Blots were analyzed by use of an enhanced chemiluminescence (ECL) system (Pierce Biotechnology, Rockford, U.S.A.) with GAPDH serving as an internal control.

### Subcellular fractionation assay

The nuclear and cytoplasmic fractions of HCC cells were separated using a PARIS kit (Invitrogen). Cytoplasmic and nuclear fractions were determined by qRT-PCR.

### Fluorescence *in situ* hybridization

HCC cells were fixed in 4% formaldehyde for 20 min and washed with PBS. Incubated with hybridization buffer mixed with Fluorescence *in situ* hybridization (FISH) probe (RiboBio). Hoechst was then applied to stain the nuclei. Finally, pictures were captured with a microscope.

### RNA pull-down assay

RNA pull-down assay was used to detect potential binding capacity between FAM83A-AS1 and NOP58. In short, biotinylated FAM83A-AS1 sense and FAM83A-AS1 antisense were respectively cultured with cell lysate from HCC cells followed by incubation with streptavidin agarose beads (Invitrogen, Carlsbad, CA, U.S.A.). The proteins bound with RNA were washed by using elution buffer and detected by Western blot assay.

### Statistical analysis

Statistical analysis was performed by SAS software (version 9.2; SAS Institute, Inc., Cary, NC, U.S.A.). For continuous variables, the data were expressed as mean  ±  SD. Student’s *t* test or one-way ANOVA was used to compare difference between experimental groups, and differences with *P*-value less than 0.05 were considered as statistically significant.

## Results

### FAM83A-AS1 expression was elevated in HCC tissues and cells and deficiency of FAM83A-AS1 suppressed the progression of HCC

**Figure 1:**
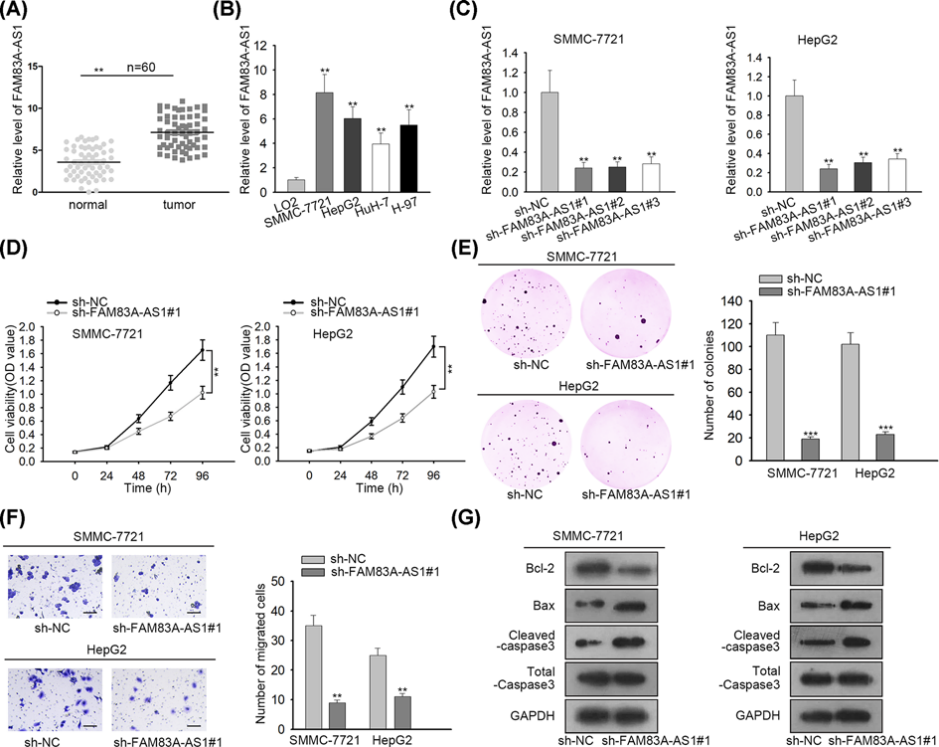
FAM83A-AS1 expression was elevated in HCC tissues and cells and deficiency of FAM83A-AS1 suppressed the progression of HCC (**A**) The expression of FAM83A-AS1 in HCC tissues and normal adjacent tissues were evaluated by qRT-PCR. (**B**) qRT-PCR detected the expression of FAM83A-AS1 in HCC cells and normal liver cells. (**C**) qRT-PCR was employed to test the knockdown efficiency of FAM83A-AS1 in HCC cells. (**D**) CCK-8 assay delineated cell proliferation ability in HCC cells. (**E**) Colony formation assay was carried out to examine the number of colonies in HCC cells. (**F**) Transwell assay was applied for estimating cell migration in HCC cells (scale bar = 200 μm). (**G**) Western blot assay was conducted to explore cell apoptosis in HCC cells. **P<0.01, ***P<0.001.

In order to scrutinize the role of FAM83A-AS1 in HCC, total RNA from HCC tissues and normal adjacent tissues was subjected to qRT-PCR, showing that FAM83A-AS1 expression was severely boosted in HCC tissues (Figure 1A). Likewise, HCC cells (SMMC-7721, HepG2, HuH-7 and H-97) also exhibited higher expression of FAM83A-AS1 than in normal liver cells (LO2) (Figure 1B). As SMMC-7721 and HepG2 cells presented higher expression of FAM83A-AS1, thus they were selected for the subsequent assays. Afterward, FAM83A-AS1 interfered in HCC cells transfected with sh-FAM83A-AS1#1/2/3 plasmids; all of them disclosed satisfactory knockdown efficiency with sh-FAM83-AS1#1 plasmid, which decreased the expression of FAM83A-AS1 most, was the best for the following assays (Figure 1C). Thereafter, a series of loss of function assays were performed to uncover the function of FAM83A-AS1 in HCC. CCK-8 assay was applied to examine cell proliferation in HCC cells, presenting that cell proliferation was strongly alleviated due to FAM83A-AS1 inhibition (Figure 1D). Similarly, colony formation assay showed that the number of colonies in HCC cells was weakened by FAM83A-AS1 repression as well (Figure 1E). Furthermore, transwell assay manifested that FAM83A-AS1 attenuation blocked cell migration in HCC cells (Figure 1F). Finally, with the employment of Western blot, protein levels of cell apoptosis biomarkers were detected. The result showed that the protein expression of Bcl-2 was decreased while the protein expression of Bax and cleaved caspase-3 was increased by FAM83A-AS1 repression (Figure 1G), indicating that FAM83A-AS1 depletion strengthened cell apoptosis in HCC cells. Hence, our results implied that FAM83A-AS1 expression was elevated in HCC tissues and cells and the deficiency of FAM83A-AS1 suppressed the progression of HCC.

### FAM83A-AS1 could positively regulate the expression of FAM83A in HCC

**Figure 2:**
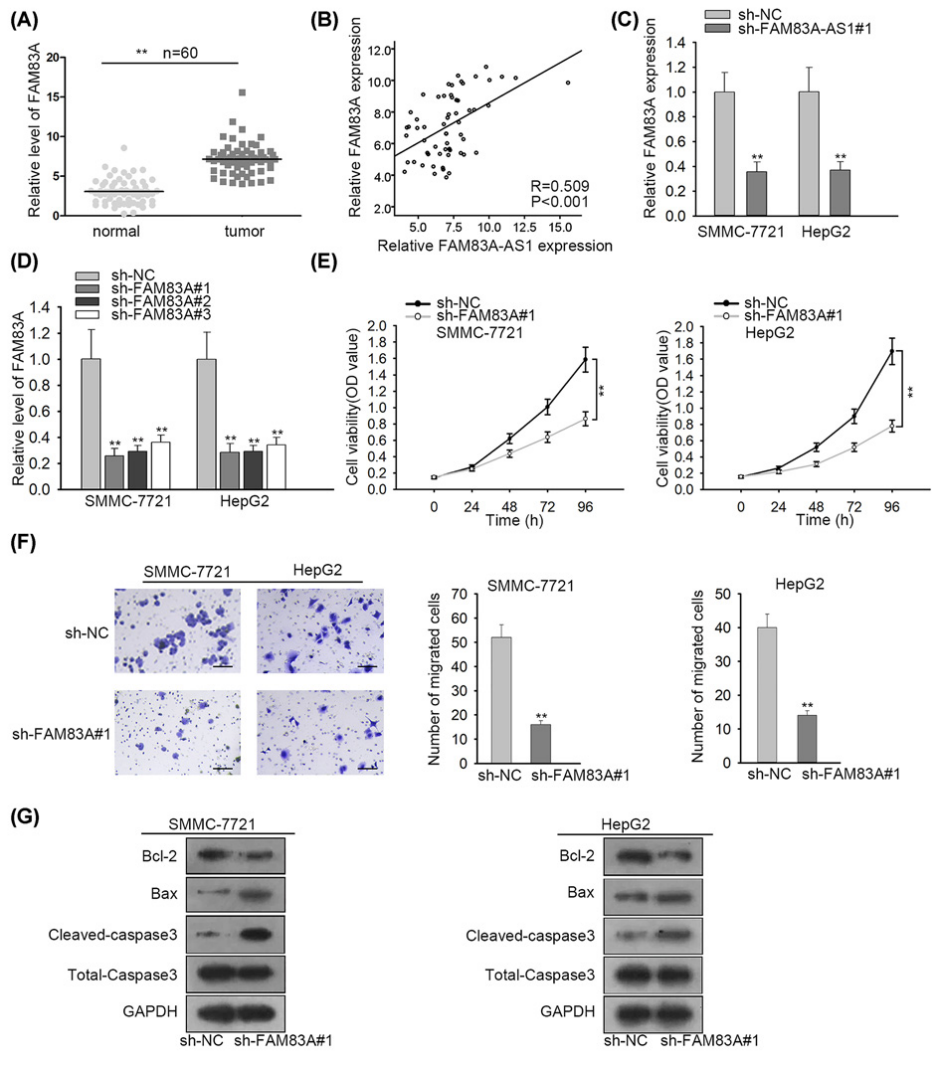
FAM83A-AS1 could positively regulate the expression of FAM83A in HCC (**A**) qRT-PCR was adopted to investigate the expression of FAM83A in HCC tissues and normal adjacent tissues. (**B**) Spearman’s correlation analysis disclosed the correlation between FAM83A-AS1 and FAM83A. (**C**) qRT-PCR was performed to detect the relationship between FAM83A-AS1 and FAM83A. (**D**) The knockdown efficacy of FAM83A in HCC cells was probed by qRT-PCR. (**E**) Cell proliferation ability in HCC cells was tested by CCK-8 assay. (**F**) Cell migration ability in HCC cells was evaluated by transwell assay (scale bar = 200 μm). (**G**) Western blot assay was applied to determine cell apoptosis ability in HCC cells. **P<0.01.

To further probe into how FAM83A-AS1 functions in HCC, the interaction between FAM83A-AS1 and FAM83A was then investigated, as there was a sequence complementarity to FAM83A with FAM83A-AS1. As Figure 2A exposed, FAM83A expression in HCC tissues was apparently increased. Besides, Spearman’s correlation analysis exhibited the positive correlation between the expression of FAM83A-AS1 and FAM83A (Figure 2B). Additionally, the expression of FAM83A in HCC was hindered by FAM83A-AS1 inhibition (Figure 2C). All these results suggested that FAM83A-AS1 could positively regulate the expression of FAM83A in HCC. Furthermore, sh-FAM83A#1/2/3 vectors were transfected into HCC cells which caused an evident decrease in FAM83A expression (Figure 2D). We chose sh-FAM83A#1 to perform the followed-by assays as the expression of FAM83A reduced the most in sh-FAM83#1 vector. CCK-8 assay showed cell proliferation ability was impaired because of FAM83A knockdown (Figure 2E). Transwell assay also disclosed a reduction in migrated cells attributed to loss of FAM83A (Figure 2F). At last, the protein expression of Bcl-2 was declined while Bax and cleaved-caspase3 were both increased by FAM83A suppression in SMMC-7721 and HepG2 cells transfected with sh-FAM83A#1 (Figure 2G). Taken together, FAM83A-AS1 could positively regulate the expression of FAM83A, and FAM83A promoted the progression of HCC.

### FAM83A-AS1 maintained FAM83A mRNA stability by binding to NOP58

**Figure 3:**
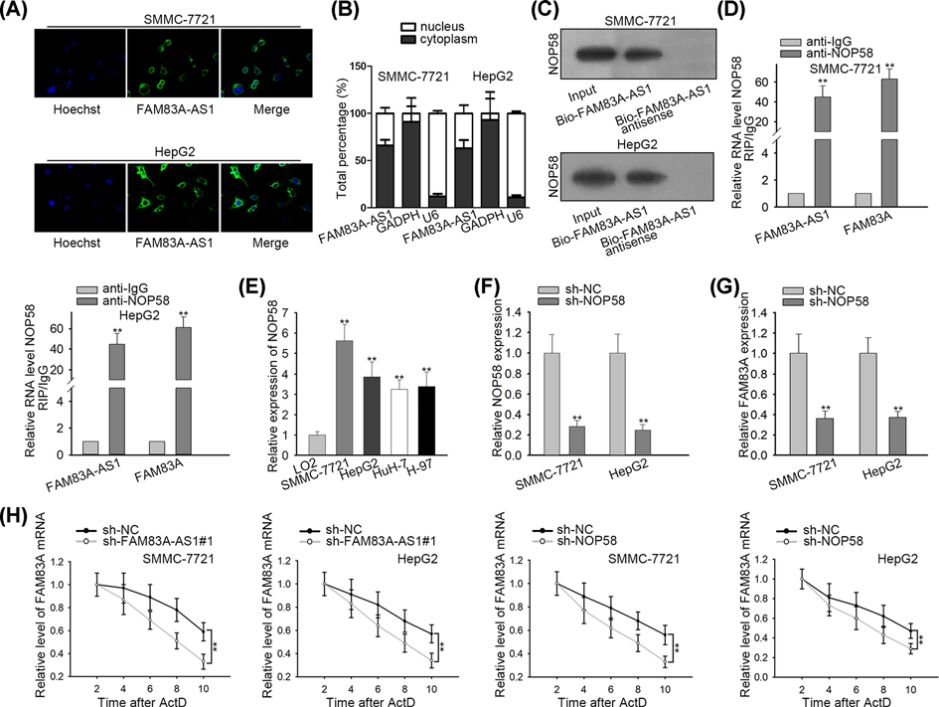
FAM83A-AS1 accelerated HCC progression by binding with NOP58 to enhance the mRNA stability of FAM83A (**A**) FISH assay was used to investigate the nuclear and cytoplasmic location of FAM83A-AS1 in HCC cells. (**B**) The nuclear and cytoplasmic location of FAM83A-AS1 in HCC cells was examined by subcellular fractionation assay. (**C**) RNA pull-down assay was used to detect the potential binding capacity of FAM83A-AS1 and NOP58. (**D**) RIP assay was used to confirm the FAM83A-AS1 and FAM83A could bind with NOP58. (**E**) The expression of NOP58 in HCC cell lines. (**F**) NOP58 was knocked down in HCC cells and its expression was examined by qRT-PCR. (**G**) The expression of FAM83A was explored by qRT-PCR. (**H**) HCC cells with FAM83A-AS1/NOP58 knockdown or not were treated with actinomycin D for the indicated times. **P<0.01.

As the relationship between FAM83A-AS1 and FAM83A was confirmed above, how FAM83A-AS1 regulated FAM83A needed to be clarified. RNA FISH and subcellular fractionation assays both demonstrated that FAM83A-AS1 was mainly located in cytoplasm (Figure 3A,B). RNA pull-down assay showed that FAM83A-AS1 could bind with NOP58 (Figure 3C). RIP assay showed FAM83A-AS1 and FAM83A were both enriched in anti-NOP58 group rather than anti-IgG group (Figure 3D), which further illustrated that FAM83A-AS1 could bind with NOP58. Besides, qRT-PCR manifested that NOP58 was overexpressed in HCC cell lines (Figure 3E). Therefore, NOP58 was knocked down in HCC cells, which presented remarkable decrease in NOP58 expression (Figure 3F). qRT-PCR showed that NOP58 deficiency resulted in a down-regulation of FAM83A expression (Figure 3G). Moreover, the mRNA stability of FAM83A was not only decreased by FAM83A-AS1 depletion but also by NOP58 suppression (Figure 3H). In summary, FAM83A-AS1 maintained FAM83A mRNA stability by binding to NOP58.

### FAM83A-AS1 accelerated HCC progression by binding with NOP58 to enhance the mRNA stability of FAM83A

**Figure 4:**
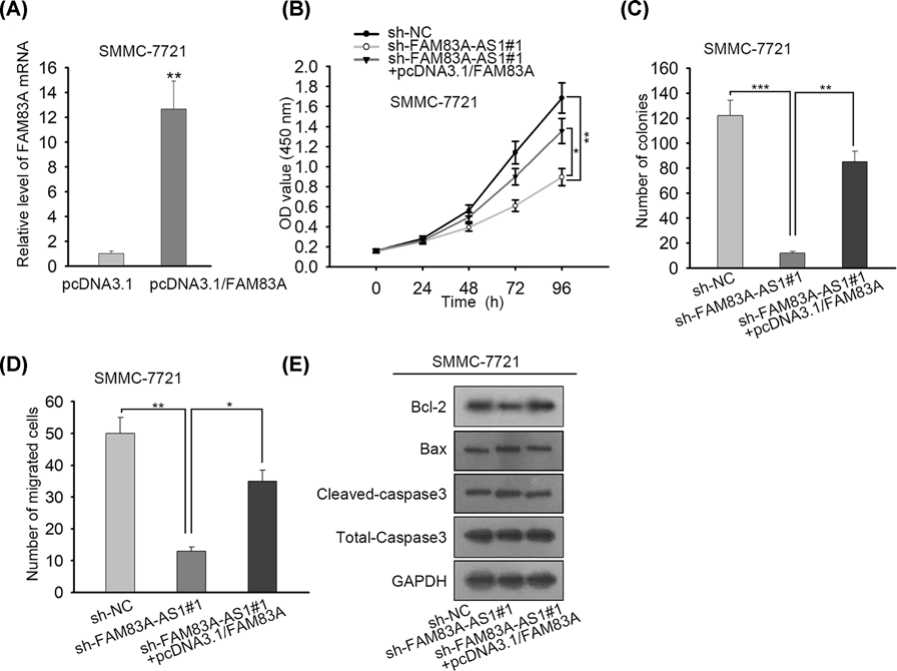
FAM83A-AS1 maintained FAM83A mRNA stability by binding to NOP58 (**A**) FAM83A was overexpressed in SMMC-7721 cells. (**B**) By utilizing CCK-8 assay, cell proliferation ability in HCC cells was inspected. (**C**) The number of colonies in HCC cells was estimated by colony formation assay. (**D**) Transwell assay showed cell migration capacity in HCC cells. (**E**) Cell apoptosis in HCC cells was explored by Western blot assay. *P<0.05, **P<0.01, ***P<0.001.

Finally, rescue assays were carried out. FAM83A was overexpressed in SMMC-7721 cells which resulted in an overt enhancement of FAM83A expression (Figure 4A). CCK-8 assay manifested that FAM83A overexpression partly countervailed FAM83-AS1 inhibition that caused decrease in cell proliferation in HCC cells (Figure 4B). Colony formation assay disclosed that overexpression of FAM83-AS1 partially offset the suppressed effect by sh-FAM83-AS1 transfection on colonies in HCC cells (Figure 4C). Furthermore, FAM83A overexpression could partly remedy the inhibitory influence of FAM83A-AS1 depletion on cell migration in HCC cells (Figure 4D). FAM83A overexpression also partially rescued the promoting effect due to FAM83A-AS1 silence on cell apoptosis in HCC cells (Figure 4E). All in all, FAM83A-AS1 accelerated HCC progression by binding with NOP58 to enhance the mRNA stability of FAM83A.

## Discussion

HCC is a prevalent malignant tumor that poses a serious threat to human health and life. High rate of recurrence is mainly owing to metastasis [14]. The carcinogenesis of HCC is complex which includes coefficient function of multiple genetic and epigenetic alterations. Accumulating researchers have proposed that numerous lncRNAs are associated with various biological processes and diseases, including cancer progression [15]. Previous evidence has also proven that the expression of lncRNAs is dysregulated in a variety of cancers, and these aberrant lncRNAs may be involved in the development of cancer progression [16–18]. However, the role of the majority of lncRNAs in cancers remains unclear. Thanks to great efforts made by a large number of researchers, abundant lncRNAs have been identified to be involved in HCC. For example, lncRNA CDKN2BAS accelerates metastasis in HCC by sponging miR-153-5p and targeting ARHGAP18 [19]. LINC01287/miR-298/STAT3 feedback loop modulates cell growth and the epithelial-to-mesenchymal transition in HCC [20]. LINC01433 facilitates the development of HCC by regulating the miR-1301/STAT3 axis [21]. Nevertheless, the role of FAM83A-AS1 in HCC is largely unknown. In current study, the function and mechanism of FAM83A-AS1 in HCC were profoundly investigated. The expression of FAM83A-AS1 was noticeably enhanced in HCC tissues and cells. Depletion of FAM83A-AS1 dramatically decreased cell proliferation and migration as well as significantly increased cell apoptosis in HCC cells. All these data illustrated that FAM83A-AS1 aggravated the progression of HCC.

FAM83A, whose expression is up-regulated in cancers, often functions as an oncogenic gene in tumorigenesis. For example, FAM83A expression is up-regulated in pancreatic cancer and promotes the progression of pancreatic cancer [22]. FAM83A contributes to HER2-positive breast cancer cells growth and inhibits cell apoptosis [23]. Moreover, FAM83A-AS1 was found to promote lung cancer cell progression via increasing FAM83A [13]. FAM83A-AS1 shared a complementary sequence with FAM83A, which provided the possibility to regulate the mRNA of FAM83A. However, the mechanism by which FAM83A-AS1 modulates FAM83A in HCC had not been reported and particularly required further exploration. In the present study, FAM83A expression was amplified in HCC tissues. FAM83A-AS1 positively regulated the expression of FAM83A. FAM83A silence inhibited cell proliferation, migration and induced cell apoptosis in HCC cells. All the data indicated that FAM83A-AS1 positively regulated FAM83A in HCC.

RNA-binding proteins (RBPs) are a group of proteins that can directly bind to RNAs [24]. Although a large number of RBPs has been identified so far, their functional investigation has fallen behind. Previously, several RBPs have been confirmed to be implicated in the progression of cancers [25,26]. In present study, RNA pull-down and RIP assays discovered that NOP58 could interact with FAM83A-AS1. NOP58 suppression decreased the expression of FAM83A. These findings illustrated that FAM83A-AS1 could recruit NOP58 to maintain FAM83A mRNA stability. Rescue assays disclosed that FAM83A partially recovered FAM83A-AS1 depletion-mediated inhibition of HCC progression.

In conclusion, our research proved that FAM83A-AS1 promoted HCC progression via binding with NOP58 to elevate the mRNA stability of FAM83A, which provides a potential therapeutic target for HCC.

## Abbreviations

AFAPI: Actin filament-associated protein 1
CCK-8: Cell Counting Kit-8
ceRNA: competing endogenous RNA
DEME: Dulbecco’s modified Eagle medium
FAM83A-AS1: family with sequence similarity 83 member A antisense RNA 1
FBS: fetal bovine serum
FISH: fluorescence *in situ* hybridization
GAPDH: glyceraldehyde-phosphate dehydrogenase
lncRNA: long noncoding RNA
NOP58: nucleolar protein 58
qRT-PCR: quantitative real-time polymerase chain reaction
RBP: RNA-binding protein
RIP: RNA immunoprecipitation
RIPA: radioimmunoprecipitation assay
SNHG15: small nucleolar RNA host gene 15
YAP1: Yes-associated protein 1
